# Supplementary material for: Characterisation of lmx1b paralogues in zebrafish reveals divergent roles in skeletal, kidney and muscle development
Source: Biol Open. 2025 Aug 19;14(8):bio062038. doi: 10.1242/bio.062038 (PMC12403520; doi:10.1242/bio.062038)
Supplement: Supplementary information [file biolopen-14-062038-s1.pdf]

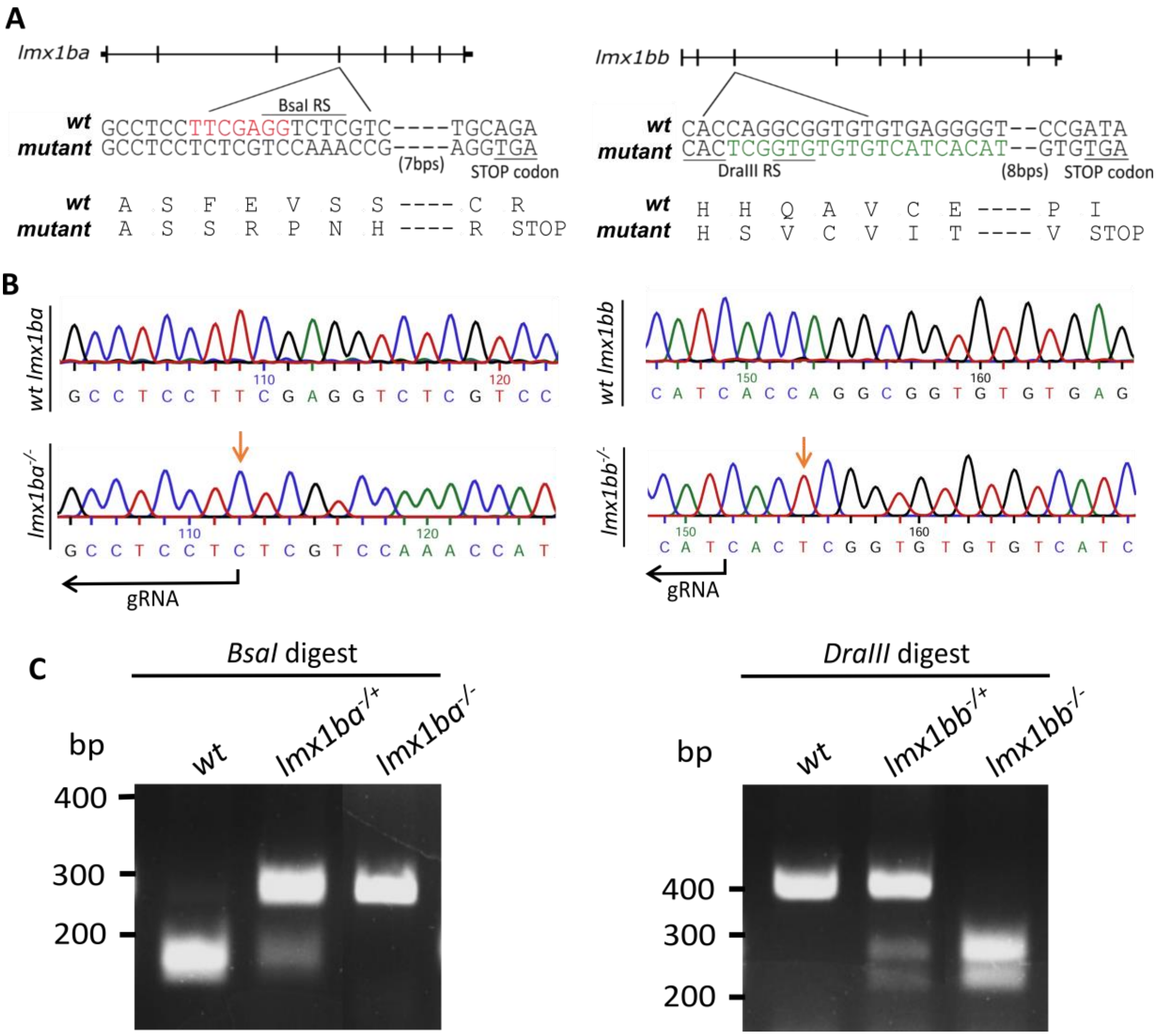

Fig. S1. *lmx1ba* and *lmx1bb* null fish lines generated using CRISPR-Cas9. (A) Schematic showing exon position of selected mutations in both *lmx1ba* and *lmx1bb* populations, with resulting nucleotide deletion or insertion depicted in red and green, respectively, and subsequent amino acid changes leading to a stop codon. These nucleotide changes result in loss of the *BsaI* restriction site in *lmxba*$^{-/-}$ and gain of the *DraIII* restriction site in *lmx1bb*$^{-/-}$. Location and sequence of restriction sites used to genotype each population highlighted. RS, restriction site. **(B)** Representative *wt* and homozygous DNA traces of *lmx1ba* and *lmx1bb* from Sanger sequencing of DNA from F2 population larvae. Beginning of mutated sequences highlighted by orange arrow. For *lmx1bb* sequences, primer set 1 was used. **(C)** Example gel images showing restriction digest of *lmx1ba* and *lmx1bb* DNA with *BsaI* and *DraIII* restriction enzymes, respectively, to separate the genotypes of each population. *DraIII* restriction digest only possible with *lmx1bb* primer set 1.

Biology Open • Supplementary information

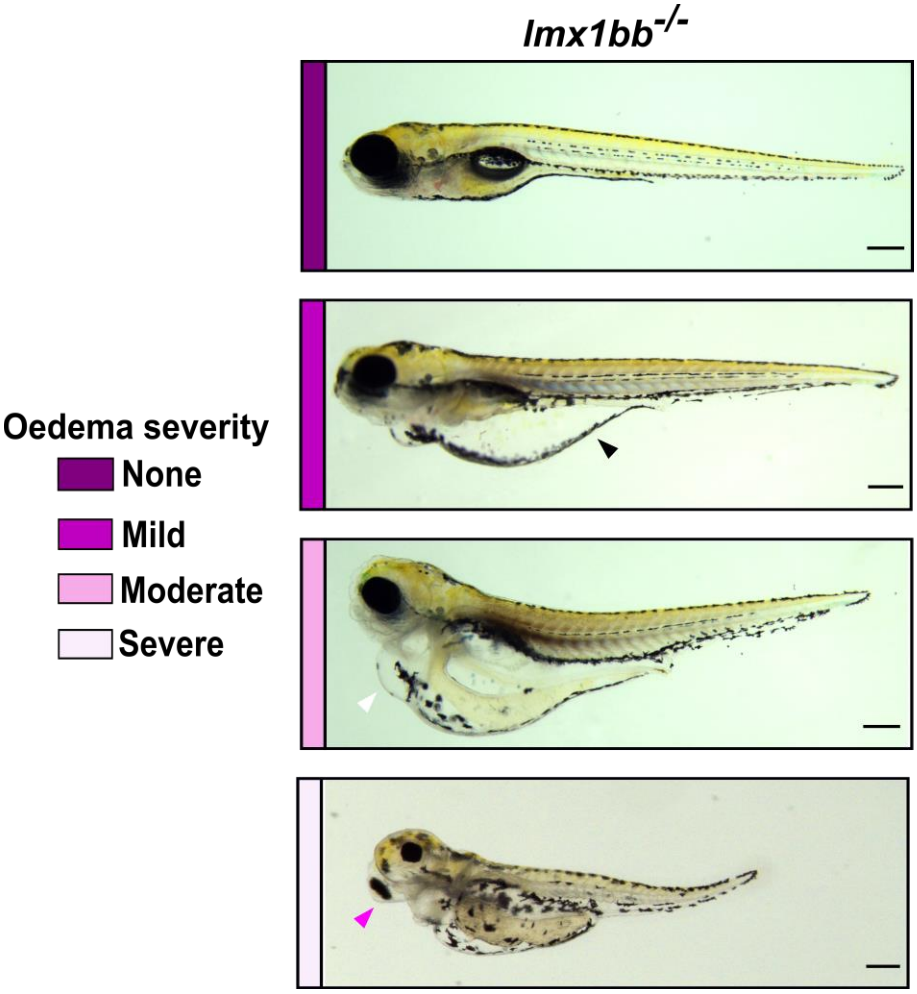

Fig. S2. *lmx1bb* mutants show range of oedema severity at 5dpf.
Stereomicroscope images of *lmx1bb* mutant larvae at 5dpf showing different severities of oedema. Mild oedema detected primarily as yolk sac oedema (*black arrowhead*); moderate oedema classified by onset of pericardial oedema (*white arrowhead*); severe oedema indicated by addition of eye oedema (*pink arrowhead*). Scale bar = 250µm.

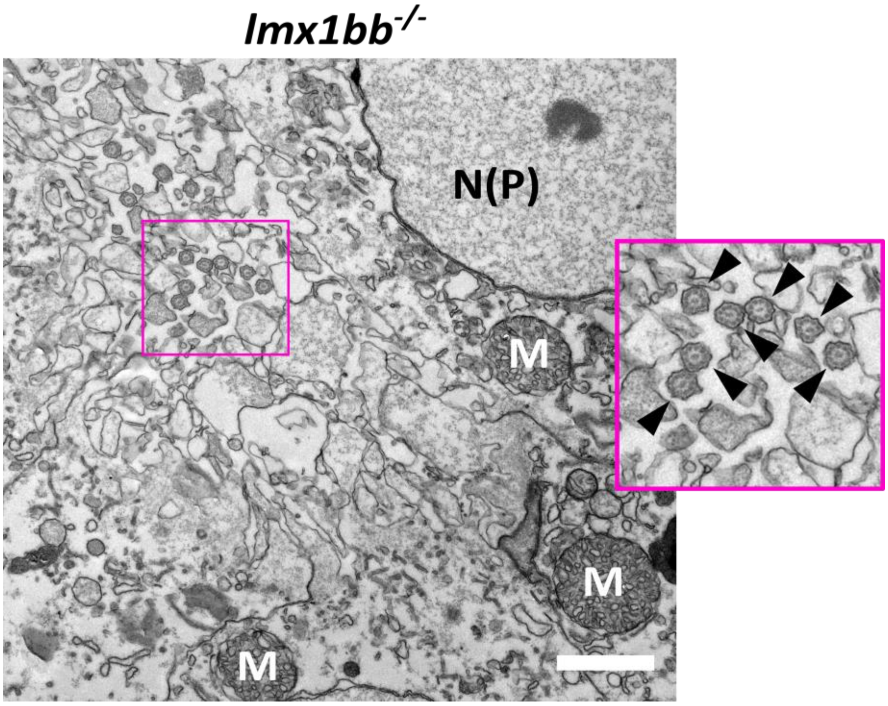

Fig. S3. Electron micrograph image of the glomeruli of a *lmx1bb* mutant at 6dpf. In some areas, the *lmx1bb* mutants show complete loss of glomerular structure and organisation and swollen mitochondria. Inset shows accumulation of motile cilia (*black arrowheads*) in *lmx1bb* mutant which could be due to reduced kidney function. M = mitochondria; N(P) = nucleus of podocyte cell. Scale bar = 500nm.

Table S1. Otolith number is unaffected, but morphology is altered in *lmx1b* mutants compared to *wt* at 5dpf.

	Total fish	Otolith no.			Normal?		Normal as a %	
		**2**	**1**	**0**	**Y**	**N**	**Y**	**N**
wt	19	19	0	0	19	0	100%	0%
lmx1ba $^{-/-}$	28	28	0	0	27	1	96%	4%
lmx1bb $^{-/-}$	17	17	0	0	10	7	59%	41%
dKO	23	23	0	0	11	12	48%	52%

Biology Open • Supplementary information
